# Arthroscopy‐controlled medial reefing and lateral release for recurrent patellar dislocation: clinical, radiologic outcomes and complications

**DOI:** 10.1186/s12891-021-04300-x

**Published:** 2021-05-10

**Authors:** Kyung Wook Nha, Hyung Suh Kim, Sung Tan Cho, Ji Hoon Bae, Ki-Mo Jang, Sang-Gyun Kim

**Affiliations:** 1 Department of Orthopaedic Surgery, Inje University Ilsan Paik Hospital, 170, Juhwa-ro, Ilsanseo-gu, Gyeonggi-do Goyang-si, 10380 Republic of Korea; 2 Department of Orthopaedic Surgery, Korea University Guro Hospital, 148, Gurodong-ro, Guro-gu, Seoul, 08308 Republic of Korea; 3 Department of Orthopaedic Surgery, Korea University Anam Hospital, 73, Goryeodae-ro, Seongbuk-gu, Seoul, 02841 Republic of Korea; 4 Department of Orthopaedic Surgery, Korea University Ansan Hospital, 123, Jeokgeum-ro, Danwon-Gu, Gyeongki-do Ansan-si, 15355 Republic of Korea

**Keywords:** Patellar dislocation, Arthroscopy‐controlled medial reefing, Kujala score, Congruence angle, Patellar tilt angle, Complication

## Abstract

**Background:**

Few studies have reported the clinical outcomes of the medial reefing procedure and lateral release with arthroscopic control of medial retinacular tension in patients with recurrent patellar dislocation. The purpose of this study was to investigate the clinical, radiologic outcomes and complications of arthroscopy-controlled medial reefing and lateral release.

**Methods:**

Patients who underwent arthroscopy-controlled medial reefing and lateral release for recurrent patellar dislocation between November 2007 and June 2017 were retrospectively evaluated. The clinical outcome (Kujala score), radiologic outcome (congruence and patellar tilt angles), and complications were evaluated at final follow-up. The results were also compared with literature-reported outcomes of other surgical procedures for patellar dislocation.

**Results:**

Twenty-five patients (mean age, 18.3 ± 4.8 years) were included in the study. The mean clinical follow-up period was 7.0 ± 2.5 (range, 3.8–12.2) years. The mean Kujala score was significantly improved from 54.7 ± 14.0 (range, 37–86) preoperatively to 91.0 ± 7.6 (range, 63–99) at a mean follow-up period of 7 years (*P* < 0.001). The radiologic results also significantly improved from 17.8° ± 5.9° to 6.8° ± 2.4° (*P* < 0.001) in the congruence angle and from 17.5° ± 8.2° to 5.6° ± 3.1° (*P* < 0.001) in the patella tilt angle at a mean follow-up period of 3.6 years. One patient developed a redislocation after a traumatic event, and two patients showed patellofemoral osteoarthritis progression.

**Conclusions:**

Arthroscopy-controlled medial reefing and lateral release significantly improved the clinical and radiologic outcomes of the patients with recurrent patellar dislocation at a mean follow-up period of 7 years. The results of this study are comparable with the literature-reported outcomes of other surgical procedures for patellar dislocation.

**Level of evidence:**

Level IV, retrospective therapeutic case series.

## Background

Patellar dislocation is one of the most common acute knee injuries in pediatric and adolescent patients [1–3]. The incidence of patellar instability is reported to be as high as 23.2 per 100,000 person-years in the United States [4]. The pathophysiology of patellar dislocation is multifactorial, and patients often have the anatomic predisposing factors for recurrent dislocation (patella alta, trochlear dysplasia, increased tibial tuberosity-trochlear groove [TT-TG] distance, femoral torsion, or genu varum deformity) [3, 5–7].

 Historically, patients with recurrent patellar dislocation have been treated with various surgical techniques such as medial patellofemoral ligament (MPFL) reconstruction and medial soft tissue realignment surgery [8–10]. A previous systematic review reported that medial soft tissue realignment surgery is associated with less favorable clinical outcomes than MPFL reconstruction [11]. However, numerous surgical techniques have been used for medial soft tissue realignment surgery. Therefore, it is not reasonable that all medial soft tissue realignment surgery is considered less valuable than MPFL reconstruction.

In this observational cohort study, patients with recurrent patellar dislocation were treated with a medial reefing procedure to attain enough tensions of the medial retinaculum and vastus medialis oblique (VMO). Moreover, the medial retinacular tension was controlled on the basis of the arthroscopic findings. To the best of our knowledge, only a few studies have reported the clinical outcomes of the medial reefing procedure and lateral release under arthroscopic control of medial retinacular tension.

The purposes of this study were to investigate the clinical, radiologic outcomes and complications of arthroscopy-controlled medial reefing and lateral release in patients with recurrent patellar dislocation. We hypothesized that arthroscopy-controlled medial reefing and lateral release would improve the clinical and radiologic outcomes with a low complication rate and that the results would be comparable with the literature-reported outcomes of other surgical procedures for patellar dislocation.

## Methods

 After receiving approval from our institutional review board, we retrospectively reviewed all the medical records and radiologic and arthroscopic data of 50 patients who underwent arthroscopy-controlled medial reefing and lateral release between November 2007 and June 2017. The surgical indication was recurrent patellar dislocation without bony abnormalities such as patella alta (Caton-Deschamps index, > 1.2), a TT-TG distance > 20 mm, and tibiofemoral valgus > 5°. The inclusion criteria were as follows: (i) primary arthroscopy-controlled medial reefing and lateral release, (ii) minimum radiologic follow-up of 1 year, and (iii) minimum clinical follow-up of 2 years. The exclusion criteria were as follows: (i) combined tibial tubercle osteotomy, (ii) combined distal femoral osteotomy, (iii) insufficient medical or radiologic data, iv) < 1-year radiologic follow-up, and v) < 2-year clinical follow-up.

### Surgical technique and rehabilitation

**Fig. 1:**
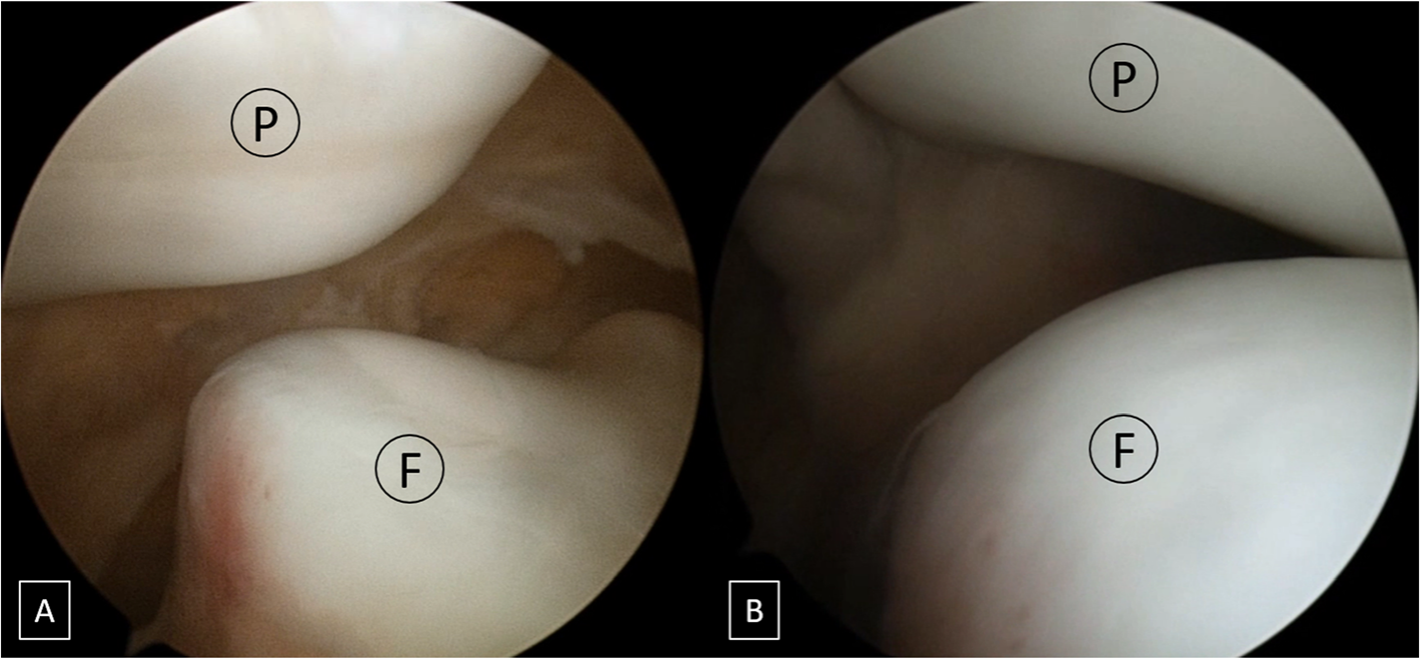
Arthroscopic findings from the superolateral portal of the left knee showing preoperative (**a**) and postoperative (**b**) statuses of patellar subluxation. **a** Patellar lateral subluxation and patellofemoral malalignment. **b** Lateral patellar edge positioned in line with the lateral trochlear border after arthroscopy-controlled medial reefing and lateral release. P, patella; F, femur

All the operations were performed by a single experienced surgeon. Diagnostic arthroscopy was performed using standard anteromedial and anterolateral portals to evaluate possible chondral lesions and concomitant pathologies. Unstable osteochondral lesions were planned to be fixed or removed, whereas stable lesions were left in situ. Then, an additional superolateral portal was established for evaluating the patellar subluxation. With the arthroscope advanced through the superolateral portal, the subluxation (or reduction) status of the patella was well visualized (Fig. 1a and b).

**Fig. 2:**
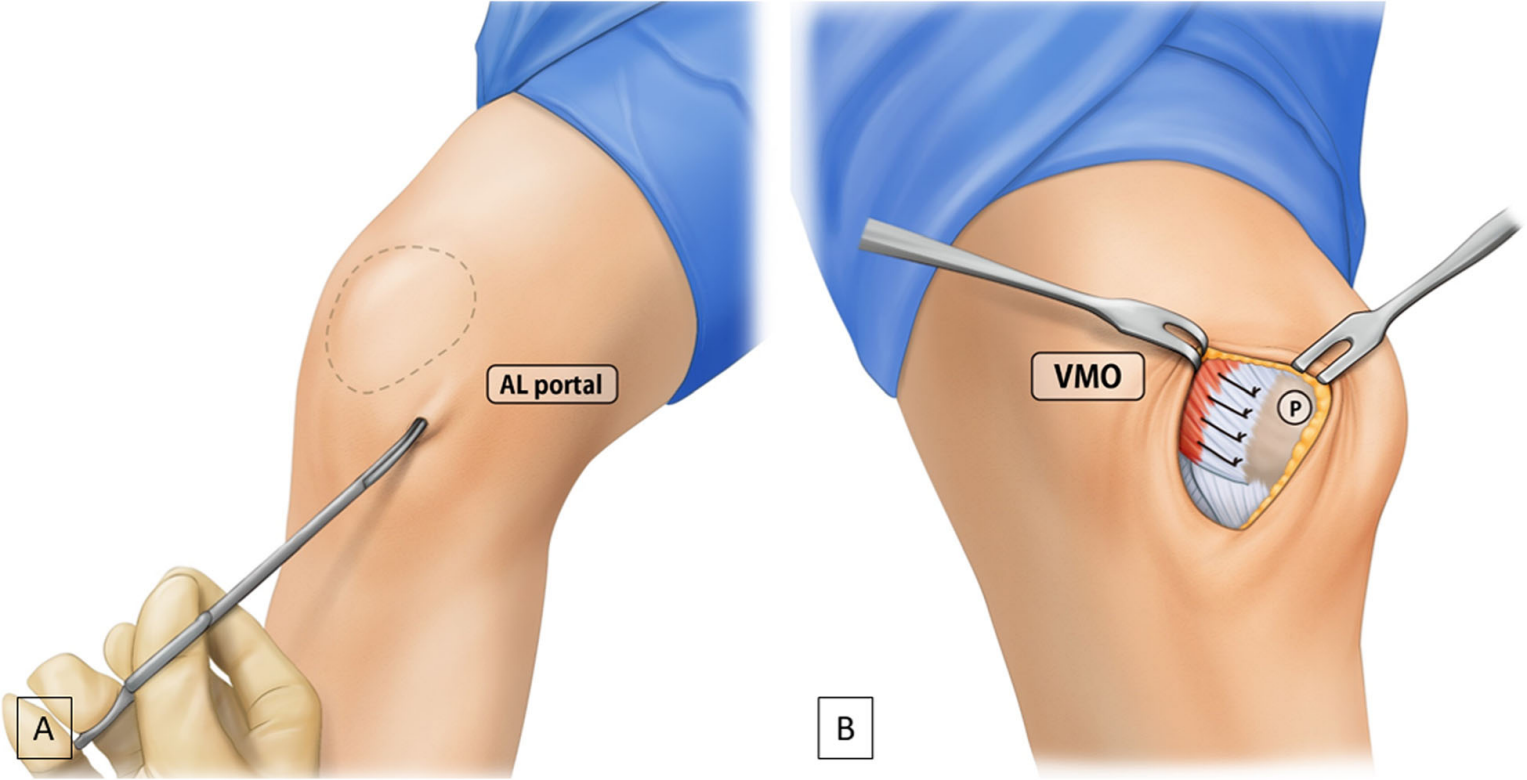
Surgical procedures of the percutaneous lateral release and medial reefing for recurrent patellar dislocation of the left knee. **a** Percutaneous lateral release using Metzembaum scissors. **b** The vastus medialis oblique muscle and medial retinacular complex advanced over the quadriceps tendon and border of the patella, with 5–10 mm of overlap depending on the arthroscopic finding of patellar reduction. VMO, vastus medialis oblique muscle

After all the arthroscopic procedures, percutaneous lateral retinacular release was performed in all patients to prevent arthritic change by reducing the patellofemoral joint pressure [12]. This release procedure was performed as previously described by Randal et al. [13]. Long Metzenbaum scissors were used to create a tunnel beneath the skin overlying the level approximately 3 to 4 cm above the lateral pole of the patella. Then, both layers of the retinaculum and synovium were transected along the lateral side of the patella to a level approximately 4 cm above the superior pole (Fig. 2a).

**Fig. 3:**
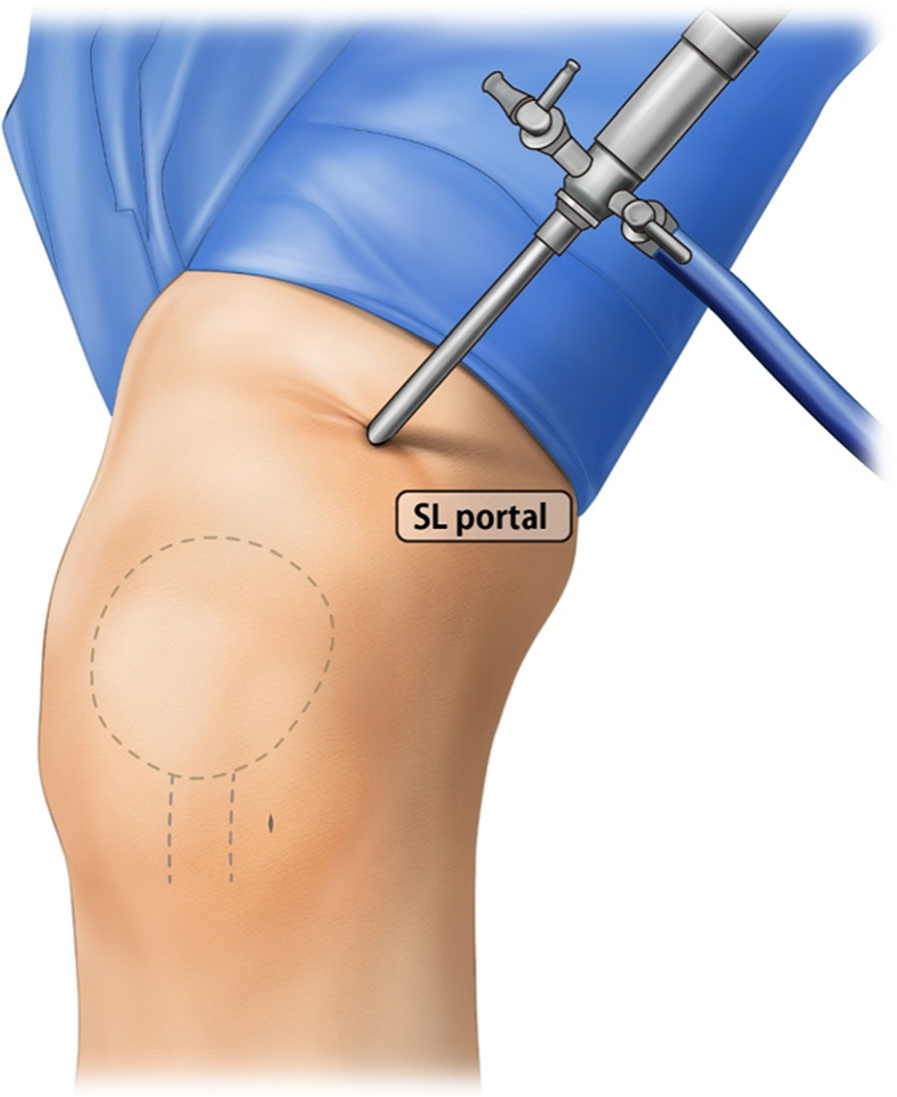
Arthroscopy-controlled evaluation of medial retinacular tension of the left knee from the superolateral (SL) portal before the mattress sutures were tied

Next, the medial reefing procedure was performed (Fig. 2b). A 5-cm longitudinal incision was made on the medial aspects of the patella. After the meticulous dissection through the subcutaneous tissues, the VMO muscle and medial retinacular complex were identified. Then, the medial retinacular complex was incised from 3 cm above the patellar upper pole to the inferior margin of the medial retinacular complex, as in medial parapatellar arthrotomy. Using No. 5 Ethibond sutures (Ethicon Inc, Johnson & Johnson, Somerville, NJ), the VMO and medial retinacular complex were advanced over the quadriceps tendon and the medial border of patella with an overlapping edge. Before the sutures were tied, the arthroscope was reintroduced into the superolateral portal to evaluate the tension of the medial retinaculum and reduction status of the patella (Fig. 3). When the lateral patellar edge was positioned in line with the lateral trochlear border, the suture tension was considered proper. On the basis of the arthroscopic finding, suture tension was either increased or decreased, as necessary. The final suture of the medial retinacular complex was performed at 30° knee flexion and in neutral rotation.

After surgery, all the patients underwent the same rehabilitation programs. Compression dressing and a cylinder splint in 30° knee flexion were applied for 3 days. Thereafter, partial weight-bearing crutch gait and tolerable range of motion exercise were allowed. Full weight bearing was started from 2 weeks after surgery.

### Outcome measurements

The clinical outcome was evaluated on the basis of the postoperative change in Kujala score. The patients’ preoperative Kujala scores were obtained before the surgical treatment, and their records were retrospectively reviewed. One senior resident, who was blinded to the study design, contacted the patients via telephone to obtain their postoperative Kujala scores.

**Fig. 4:**
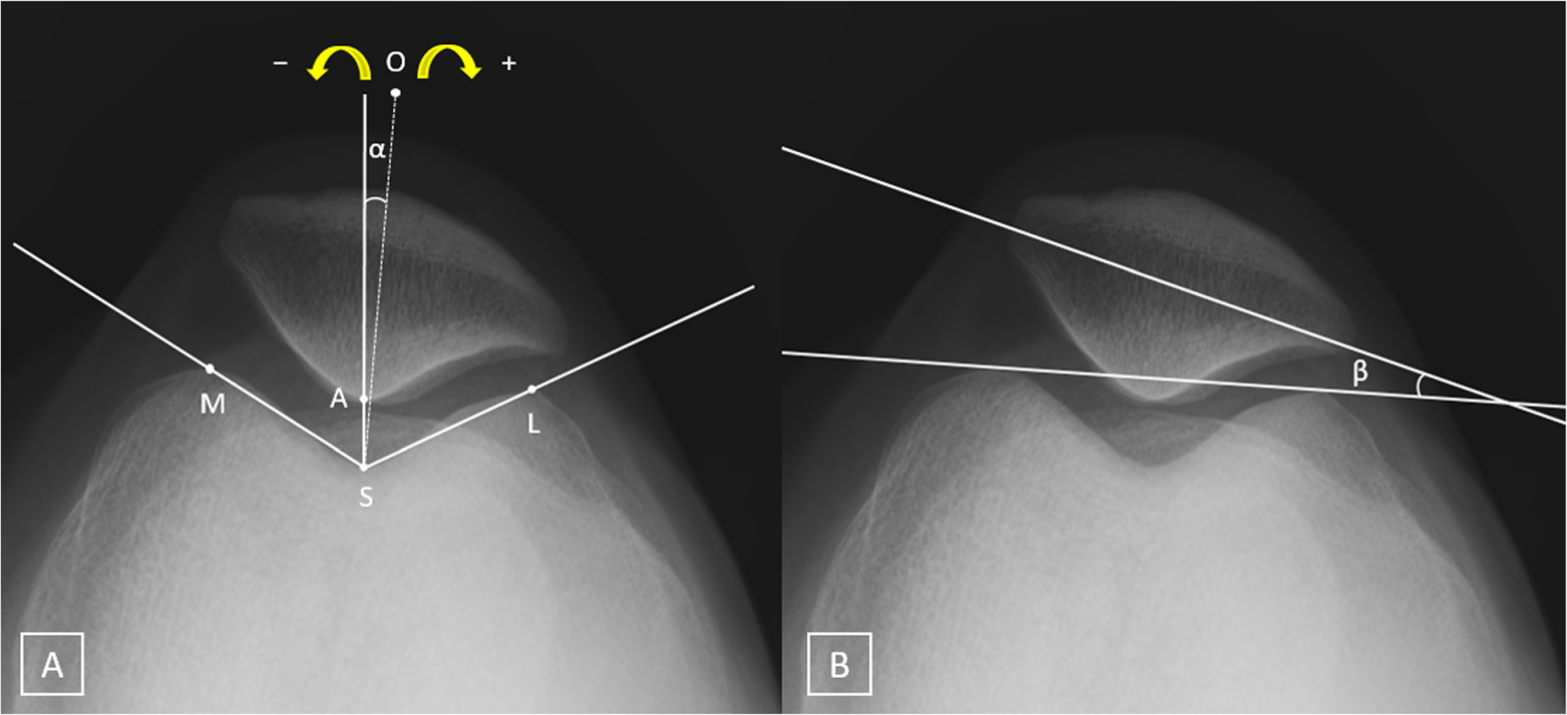
Measurement of the congruence and patellar tilt angles on the left knee skyline view (inferior-superior projection of the patella in 45° flexion). **a** Congruence angle measurement. The highest point of the medial (M) and lateral (L) condyles, and the lowest point of the intercondylar sulcus (S) were identified. The zero-reference line (SO, dotted line) bisecting the sulcus angle (MSL) was established. The lowest point on the articular ridge of the patella (A) was identified, and a line from S to A was drawn. The congruence angle was measured as the angle ASO (*α*). All values medial and lateral to the zero-reference line SO are designated as negative and positive, respectively. **b** The patellar tilt angle (*β*) was measured as the angle between a line intersecting the widest bony structure of the patella and a line tangential to the anterior surface of the femoral condyles on a skyline view

Two clinical fellows who were not involved in the study conducted radiologic evaluations using congruence and patellar tilt angles preoperatively and at the final follow-up visit (Fig. 4a and b).

Postoperative complications such as redislocation, patellar fracture, patellofemoral osteoarthritis (PFOA) progression, infection, and stiffness were compared between the groups. PFOA progression was defined as an arthritic change that is more progressive than the preoperative state of the patellofemoral joint on skyline radiography at the final follow-up [8]. Arthritic changes of the patellofemoral joint were qualitatively evaluated using the Kellgren-Lawrence (K-L) classification [14].

### Data analysis and statistical methods

All statistical analyses were performed with SPSS version 21.0 (SPSS Inc., Chicago, IL, USA). Quantitative variables are presented as mean values and standard deviations with ranges.

Paired *t*-test was used to evaluate the postoperative change in the Kujala score, congruence angle, and patellar tilt angle. The PFOA progression was evaluated using the Wilcoxon signed rank test. Statistical significance was set at a *P* value of < 0.05.

Intraobserver and interobserver reliabilities were determined by calculating the intraclass correlation coefficients (ICCs) for the radiologic outcome parameters such as congruence angle, patellar tilt angle, and PFOA (K-L grade). An ICC of < 0.40 was considered poor, whereas ICCs of 0.40–0.59, 0.60–0.74, and 0.75–1.00 were considered fair, good, and excellent, respectively [15].

## Results

**Fig. 5:**
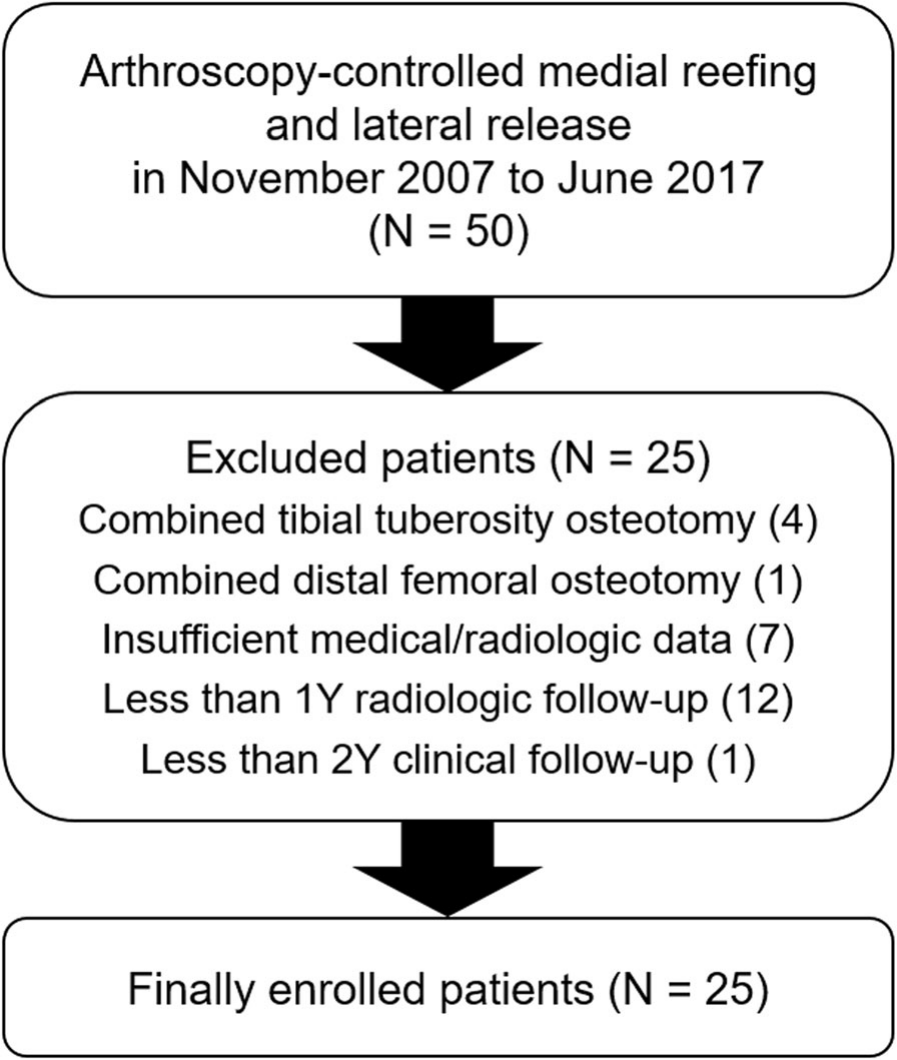
Flowchart of the enrolled patients in this study

Twenty-five patients (mean age, 18.3 ± 4.8 years) were included in the study (Fig. 5). The demographic data of the enrolled patients are summarized in Table 1. The mean clinical and radiologic follow-up periods were 7.0 ± 2.5 years (range, 3.8–12.2 years) and 3.6 ± 2.1 years (range, 1.0–8.3 years), respectively. Osteochondral lesions, which were left in situ because they were stable, were identified in 11 patients (44 %).

**Table 1:** Preoperative demographic data and radiologic evaluation

	Enrolled patients (*n* = 25)
***Preoperative demographic data***	
Age at surgery^a^, years	18.3 ± 4.8 (11.0–31.0)
Male sex, n (%)	12 (48.0)
Injury side, right, n (%)	6 (24.0)
Body mass index^a^, kg/m^2^	22.4 ± 3.8 (17.5–31.6)
Number of dislocations, n	
2–4	22
≥5	3
Interval between the onset of primary dislocation and surgery†, months	12.3 ± 25.7 (1.0–92.0)
Radiological follow-up period^a^, years	3.6 ± 2.1 (1.0–8.3)
Clinical follow-up period^a^, years	7.0 ± 2.5 (3.8–12.2)
***Preoperative radiologic evaluation***	
Hip-knee-ankle angle^a^, °	-1.9 ± 6.3 (-18.0–7.0)
Preoperative C-D index^a^	-0.9 ± 0.1 (0.7–1.1)
TT-TG distance^a^, mm	13.1 ± 2.3 (9.0–18.0)
Trochlea dysplasia, Dejour type A-B-C-D	8-5-11-1
Osteochondral fragment, n (%)	11 (44.0)

*C-D* Caton-Deschamps, *TT-TG* tibial tuberosity-trochlea groove

^a^Values are presented as mean ± standard deviation with the range in parentheses

The mean Kujala score was significantly improved from 54.7 ± 14.0 (range, 37–86) preoperatively to 91.0 ± 7.6 (63–99) at the final follow-up (*P* < 0.001). The minimum postoperative Kujala score was 84 points, except in one patient who had a redislocation. The radiologic results also significantly improved at the final follow-up, from 17.8° ± 5.9 to 6.8° ± 2.4° (*P* < 0.001) in the congruence angle and from 17.5° ± 8.2° to 5.6° ± 3.1° (*P* < 0.001) in the patella tilt angle (Table 2). All the ICC values for the radiologic outcome parameters were > 0.8.

**Table 2:** Comparison of preoperative and postoperative clinical and radiologic outcomes

	Preoperative	Postoperative	*P* value
Kujala score^a^	54.7 ± 14.0 (37–86)	91.0 ± 7.6 (63–99)	< 0.001
Congruence angle^a^, °	17.8 ± 5.9 (6.0–28.0)	6.8 ± 2.4 (3.0–14.0)	< 0.001
Patellar tilt angle^a^, °	17.5 ± 8.2 (4.0–32.0)	5.6 ± 3.1 (2.0–11.0)	< 0.001
PFOA, K-L grade 0-1-2-3-4	24-1-0-0-0	22-3-0-0-0	0.157

*PFOA* patellofemoral osteoarthritis, *K-L* Kellgren-Lawrence

^a^Values are presented as mean ± standard deviation with the range in parentheses

One patient had a redislocation after a traumatic event, after which the patient felt that the patellar dislocation had recurred. However, the patient did not want a revision surgery; thus, she was treated conservatively. PFOA progression was observed in two patients at the final follow-up of 19 and 38 month, respectively. However, no other complications such as patellar fracture, infection, stiffness, or hemarthrosis occurred (Table 3).

**Table 3:** Complications of medial reefing and lateral release

	Enrolled patients (*n* = 25)
Total complications, n (%)	3 (12.0)
Re-dislocation, n (%)	1 (4.0)
Patella fracture, n (%)	0 (0.0)
PFOA progression, n (%)	2 (8.0)
Infection, n (%)	0 (0.0)
Stiffness, n (%)	0 (0.0)
Hemarthrosis, n (%)	0 (0.0)

*PFOA* patellofemoral osteoarthritis

## Discussion

The most important finding of this study was that arthroscopy-controlled medial reefing and lateral release significantly improved the clinical and radiologic outcomes of patients with recurrent patellar dislocation. The results of this study showed excellent Kujala scores and radiologic improvements at a mean follow-up period of 7 and 3.6 years, respectively. Furthermore, only one patient had a redislocation, which resulted from a traumatic event. Therefore, the results of this study suggest that arthroscopy-controlled medial reefing and lateral release is safe and effective treatment for patients with recurrent patellar dislocation.

Previously, Nam et al. introduced mini-open medial reefing and arthroscopic lateral release for recurrent patellar dislocation (Table 4) [16]. The surgical technique they used was similar to that used in our study, but with several differences. First, Nam et al. arthroscopically released the lateral retinaculum, whereas we performed a percutaneous lateral release. Second, Nam et al. performed final sutures of the medial retinaculum in full knee extension. In arthroscopy-controlled medial reefing, the tension of the medial retinaculum was evaluated on the basis of an arthroscopic finding of patellar reduction. Medial soft tissue stabilizers (quadriceps muscle, medial retinaculum, and MPFL) mainly function as lateral patellar restraints from 0° to 30° knee flexion [17, 18]. However, the patella does not engage in the trochlea groove in < 30° knee flexion [18]; thus, the reduction status of the patella is difficult to evaluate on the basis of arthroscopic findings. For this reason, we performed the final suture of the medial retinaculum in 30° knee flexion.

**Table 4:** Present and previous studies reporting midterm clinical and radiological outcomes of medial soft tissue procedures for patellar dislocations

	Present study	Nam et al.	Cerciell et al.	Liu et al.
Journal (year)	-	AJSM (2005)	KSSTA (2014)	AJSM (2018)
Type of study	ORS	ORS	ORS	ORS
Surgical technique for medial reefing	Open (extended) reefing of medial retinacular complex and VMO under arthroscopic tension control	Mini-open reefing of medial retinacular complex and VMO under arthroscopic tension control	Percutaneous reefing of the medial retinacular complex under arthroscopic tension control	Reconstruction of the medial patellofemoral ligament
Additional procedure	Percutaneous lateral release	Arthroscopic lateral release	No	No
Number of cases	25	23	30	121
F-U period, months	83.9 (45–146)	53 (17–168)	72 (44–114)	43.7 (24–111)
Postoperative Kujala score^†^	91.0 ± 7.6	88.2 ± 13.5	88.4 ± 7.6	90.0 ± 11.8
Postoperative congruence angle^a^, °	6.8 ± 2.4	-11.5 ± 8.7	NR	NR
Postoperative patellar tilt angle^a^, °	5.6 ± 3.1	NR	9.0 ± 2.7	NR
Redislocation, n (%)	1 (4.0)	2 (8.7)	No	3 (2.5)

*AJSM* American Journal of Sports Medicine, *KSSTA* Knee Surgery, Sports Traumatology, Arthroscopy, *F-U *follow-up, *NR* not reported, *ORS* observational retrospective study, VMO

^a^Values are presented as mean ± standard deviation

The results of this study are comparable with the literature-reported outcomes of other surgical procedures for patellar dislocation (Table 4). Nam et al. reported the clinical outcomes of mini-open medial reefing and arthroscopic lateral release [16]. They reported that the mean Kujala knee score was 88.2 ± 13.5, with an overall good score in each category at a mean follow-up period of 4.4 years. Cerciell et al. introduced all-inside arthroscopic techniques for medial reefing and reported that the mean Kujala score improved to 88.4 ± 7.6 at the 6-year follow-up. On the other hand, Liu et al. reported mid-term clinical outcomes of MPFL reconstruction. In this study, the postoperative Kujala score improved to 90.0 ± 11.8, and 3 (2.5 %) of the 121 patients had a redislocation after surgery.

Currently, MPFL reconstruction is the gold standard in the surgical management of recurrent patellar dislocation without severe bony abnormality [19]. The MPFL is the most important soft tissue stabilizer against patellar dislocation from 0° to 30° knee flexion [20]. MPFL reconstruction has shown excellent mid-term and long-term clinical outcomes, with a low rate of recurrence [21, 22]. However, performing the procedure requires technical refinement, as complication rates as high as 26 % have been reported [23]. Femoral tunnel malposition may result in graft anisometry, which leads to graft laxity and, ultimately, early failure or excessive patellofemoral compression forces and arthrosis [19]. In addition, appropriate graft length and tension are essential for successful MPFL reconstruction [24]. However, it is difficult to adjust the length and tension of the graft once it is fixed in the tunnel during MPFL reconstruction.

The medial reefing procedure is a soft tissue surgery that does not require a patellar or femoral bone tunnel, unlike MPFL reconstruction. Soft tissue surgery without a bone tunnel has several advantages. Creating a bone tunnel causes additional injury to patients. It has a potential risk of growth plate injury, especially in pediatric or adolescent patients. Considering that patellar dislocation is one of the most common acute knee injuries in pediatric and adolescent patients with open growth plates [1], arthroscopy-controlled medial reefing and lateral release can be safely performed without delay of the surgical treatment in these patients. Furthermore, soft tissue surgery does not interfere with other combined surgeries such as combined ligament reconstruction or corrective osteotomy. The femoral bone tunnel in MPFL reconstruction can cause tunnel collision during combined ligament reconstruction such as posterior cruciate ligament or medial collateral ligament reconstruction. It also interferes with the osteotomy or screw fixation during distal femoral corrective osteotomy. In addition, the soft tissue procedure has no risk of patellar fracture. The transosseous tunnel for patellar fixation is a well-known risk factor of patellar fracture after MPFL reconstruction [23, 25]. Although suture anchor patellar fixation for MPFL reconstruction is a relatively safe method, a previous case report showed that it can also cause patellar fracture without traumatic events [26]. Therefore, we believe that arthroscopy-controlled medial reefing and lateral release can be considered as a viable and effective treatment option for recurrent patellar dislocation.

VMO is an important dynamic structure in preventing patella lateral translation. A previous biomechanical study reported that the tension of the VMO significantly changed the patellar stability throughout the knee range of motion [27]. In this study, relaxing the VMO tension reduced the patellar stabilizing force by 47 %. Stephen et al. also reported that decreased tension of the VMO resulted in significant increases in lateral patellar tilt and translation [28]. Therefore, the tension of the VMO plays an important role in the stability of the patellofemoral joint. However, arthroscopic medial reefing procedures may be limited in the surgical approach to advance the VMO and proximal retinaculum. Therefore, medial reefing procedures were performed with open surgery to reef the proximal retinaculum and VMO in this study. This may have influenced the satisfactory clinical outcomes and low redislocation rate at the mid-term follow-up in this study.

### Limitations

This study has several limitations. First, it was a retrospective and nonrandomized study. Furthermore, 20/50 patients (40 %) were excluded due to insufficient data or being lost to follow-up. Therefore, selection bias may have influenced the results. Second, the number of patients included in this study was too small. Additional studies with larger sample sizes are needed to make more precise conclusions. Third, in this study, the redislocation rate was assessed based on the patient’s reported outcome via a telephone survey, not objective radiography or physical examination. However, we believe that the patient could have recognized the patellar redislocation because they had often experienced pain and discomfort caused by patellar dislocation before surgery. Finally, the long-term outcomes of the arthroscopy-controlled medial reefing procedure could not be investigated in this study. Schorn et al. reported that the long-term outcomes after arthroscopic medial reefing and lateral release were not satisfactory [10]. They reported that the probability of recurrent patellar dislocation after surgical treatment over time was 16 % after 1 year and 42 % after 5 years and was increased up to 52 % at the 10-year follow-up. In the present study, only one patient (4.0 %) had a patellar redislocation at a mean follow-up period of 7 years. However, evaluation of the long-term clinical outcomes, including the redislocation rate, would be required to confirm that the tension of the medial retinacular complex could be maintained.

## Conclusions

Arthroscopy-controlled medial reefing and lateral release significantly improved the clinical and radiologic outcomes of the patients with recurrent patellar dislocation at a mean follow-up period of 7 years. The results of this study are comparable with the literature-reported outcomes of other surgical procedures for patellar dislocation.

## Data Availability

The dataset used during the current study is available from the corresponding author on reasonable request.

## Abbreviations

TT-TG: Tibial tuberosity-trochlear groove
MPFL: Medial patellofemoral ligament
VMO: Vastus medialis oblique
PFOA: Patellofemoral osteoarthritis
K-L: Kellgren-Lawrence
ICC: Intraclass correlation coefficients
